# MRI-derived estimation of biological aging in patients with affective disorders in a 9-year follow-up - a prospective marker of future recurrence

**DOI:** 10.1038/s41380-025-03382-6

**Published:** 2025-12-14

**Authors:** Katharina Förster, Nils R. Winter, Jan Ernsting, Ramona Leenings, Lukas Fisch, Carlotta Barkhau, Maximilian Konowski, Daniel Emden, Anna Kraus, Katharina Dohm, Klaus Berger, Volker Arolt, Angela Carballedo, Danutia Lisiecka, Thomas Frodl, Philipp Kanske, Udo Dannlowski, Tim Hahn, Dominik Grotegerd

**Affiliations:** 1 Child and Adolescent Psychotherapy, Institute of Psychology, Universität Hamburg, Hamburg, Germany; 2 Clinical Psychology and Behavioral Neuroscience, Faculty of Psychology, Technische Universität Dresden, Dresden, Germany; 3 Institute for Translational Psychiatry, University of Münster, Münster, Germany; 4 Institute for Geoinformatics, University of Münster, Münster, Germany; 5 Faculty of Mathematics and Computer Science, University of Münster, Münster, Germany; 6 Department of Psychiatry and Neuroscience, Charité Universitätsmedizin Berlin, Berlin, Germany; 7 Institute of Epidemiology and Social Medicine, University of Münster, Münster, Germany; 8 Department of Psychiatry & Trinity College Institute of Neuroscience, University Dublin, Dublin, Ireland; 9 Department of Psychiatry, Psychotherapy and Psychosomatics, University Hospital Aachen, RWTH University Aachen, Aachen, Germany; 10 German Center for Mental Health, CIRC Jena, Magdeburg, Halle Germany; 11 Department of Psychology, Faculty of Psychology and Educational Sciences, Babeș-Bolyai University, 37 Republicii Street, 400015 Cluj-Napoca, Romania; 12 Department of Psychiatry, Medical School and University Medical Center OWL, Protestant Hospital of the Bethel Foundation, Bielefeld University, Bielefeld, Germany

**Keywords:** Predictive markers, Neuroscience, Depression

## Abstract

We investigated whether the brain age gap (BAG)—the difference between chronological age and age estimated from structural MRI scans—is associated with long-term disease course in affective disorders, using a prospective nine-year follow-up design. T1-weighted MRI data were collected at two time points (mean interval = 8.98 ± 2.20 years) from patients with Major Depressive Disorder (MDD; N = 32), Bipolar Disorder (BD; N = 6), and healthy controls (HC; N = 37) across two sites. Using a brain age prediction model trained on a sample of over 10,000 subjects of the German National Cohort (GNC), we estimated individual BAG at baseline and follow-up using gray matter segments derived from MRI images. Employing linear-mixed-effects models, we tested main effects of diagnosis and hospitalizations as well as their interaction with time on BAG. In an exploratory analysis, we tested if BAG at baseline was predictive of hospitalizations during the nine-year follow-up using logistic regression and 10-fold nested cross-validation. MDD patients showed significantly higher BAG compared to HC (2.27 ± 5.68 vs. 1.00 ± 5.12 years, *d* = –0.23), while BAG in BD patients was descriptively elevated (4.71 ± 5.40 years). In the Münster subsample (N = 52), patients with at least one hospitalization had higher BAG than those without (4.16 ± 5.74 vs. 1.65 ± 5.41 years, *d* = –0.45). No group-by-time interaction was observed. Higher BAG at baseline predicted hospitalization during follow-up (*p* = 0.035), although cross-validated prediction accuracy (64.3%) did not reach significance (*p* = 0.071). BAG remained stable over time and was not influenced by future recurrence, supporting its role as a potential trait-like marker of vulnerability to illness recurrence. While exploratory, these findings suggest that BAG may capture individual risk for future hospitalization in affective disorders.

## Introduction

Affective disorders contribute immensely to the global burden of disease worldwide [1, 2]. While some patients do recover and are able to live with only minor health restrictions, others suffer from recurrence and report an increasing disability leading to early retirement, a reduced quality of life and elevated mortality rates [3, 4]. Consequently, affective disorders substantially contribute to years lived with disability worldwide [5].

While for many medical conditions treatment and survival rates have dramatically improved in the last decades, mortality and prevalence rates as well as standard diagnostic procedures for mental disorders have remained virtually unchanged [6]. Multiple reasons that might explain the lack of success in the discovery of neurobiological signatures and useful biomarkers have been suggested such as a lack of validity of clinical diagnoses or the heterogeneity of characteristics within patient populations with the same diagnosis, but also the primarily univariate and unidimensional statistical approach traditionally used in neuroimaging [7, 8]. To overcome these obstacles, researchers have recommended using a combination of advanced neuroimaging with machine learning methods to establish multivariate brain signatures associated with mental disorders that improve diagnostic accuracy or optimize treatment response prediction [8–10].

Recently, a novel multivariate biomarker has emerged in the field of neuroimaging, aiming to quantify the brain changes associated with aging [11]. To this end, so-called “brain age gaps” (BAG) can be calculated to estimate the “biological age” of an individual’s brain [12, 13]. First, a multivariate machine learning model is trained on a normative population to predict chronological age from structural MRI data. Second, a disease population is fed to the trained model which estimates brain age of the individual patient. Finally, BAG estimates are calculated for every patient by subtracting chronological from predicted age. Positive BAGs thus indicate an accelerated brain aging in comparison to the normative age trajectory. As the brain age approach is based on a multivariate model, it makes use of the multi-dimensionality of neuroimaging data and is therefore able to integrate all gray matter information indicative of age. This complex pattern of age-related changes is then integrated into a single biomarker. The underlying hypothesis of the brain age prediction paradigm is that this brain age gap may serve as a marker of disease risk and there are a number of studies emphasizing an association between brain age gaps and clinically relevant variables [14, 15]. For instance, a brain age model trained with gray matter segments from healthy people was predictive of a history of stroke, diabetes, and alcohol intake as well as cognitive performance in a large sample of > 14,000 individuals from the UK Biobank [16]. Other studies show an association with general mortality as well as neurological disorders such as multiple sclerosis, mild cognitive impairment (MCI) and Alzheimer’s disease [17–19]. Importantly, brain age gaps are not only associated with diagnosis per se but have been found to be predictive of the illness trajectory, predicting severity in multiple sclerosis or conversion to Alzheimer’s disease [20, 21]. Finally, brain age gaps appear to be altered not only in neurological diseases but also across mental disorders and risk factors for mental disorders, such as early life adversity [22–24]. So far, however, most studies investigate cross-sectional differences in brain age gaps between disorders. Thus, to what extent increased BAGs are a consequence or predictor of mental disorders still remains unclear, although some evidence suggests increasing BAGs after disease onset [25].

Here, we apply an independent brain age model trained on a large normative sample of over 10,000 subjects to a longitudinal sample of patients with affective disorders measured at a large time interval, averaging 9 years. We aimed to explore whether brain age estimates may serve as a potential longitudinal marker of future illness course in patients with affective disorders.

1. We expect that patients with affective disorders show an increased BAG compared to HC (main effect diagnosis, diagnosis model).
2. Given that decreases in gray matter volume have previously been linked to greater cumulative illness severity indicated, for example, by an increased number of hospitalizations in affective disorders [26–30], we expect patients with a recurrent disease course between scans, that is having new hospitalizations, to show higher BAGs than patients without further hospitalizations and healthy controls (main effect hospitalization, hospitalization model).
3. Based on findings indicating treatment-related increases in gray matter volume [31–33] and reductions in BAG estimates [34], we expect patients with a recurrent disease course in the interval to show an increase in BAG at follow-up, while patients who recover over time will show a decrease in BAG over time (course of disease by time interaction, hospitalization model).

## Methods

### Participants and procedure

A total of 87 participants were initially investigated. N = 75 individuals remained for the analysis of the data after MRI preprocessing and quality control (Dublin: N = 23, Münster: N = 52). In Münster, 28 patients with affective disorders (BD = 6, MDD = 22) and healthy controls (N = 24) were investigated at follow-up between 8 and 12 years after their initial recruitment. For further information see our recent publication on longitudinal gray matter changes in the same sample [28]. All participants provided informed consent. Procedures were approved by the local ethics committee at the Faculty of Medicine, University of Münster and the Trinity College Dublin. There was no overlap between the participants taking part in our study and the German National Cohort (GNC) whose participants’ data was used for the development of the brain age prediction model.

### Material

#### Course of disease

Patients who reported no hospitalization between scans were categorized as “nonhospitalized” (N = 15). Patients who reported a hospitalization between scans were categorized as “hospitalized” (N = 13). The analysis was restricted to the Münster subsample because all patients in the Dublin sample received outpatient treatment (see Tables 1, 2 as well as Supplemental Material S2).

**Table 1:** Sample characteristics of the Münster-Dublin Longitudinal Cohort.

	Healthy Controls (*N* = 37) *M* (*SD*)		MDD patients (*N* = 32) *M* (*SD*)		BD patients (*N* = 6) *mean* (*SD*)		Group ANOVA	
	Baseline	Follow Up	Baseline	Follow Up	Baseline	Follow Up	*p*-value	Post hoc
Age	33.73 (12.97)	42.65 (12.63)	37.66 (10.62)	46.23 (9.87)	34.00 (7.51)	44.33 (7.06)	0.386	-
BAG	1.97 (5.10)	0.04 (5.93)	3.19 (5.43)	1.35 (5.85)	6.03 (5.69)	3.38 (5.25)	-	
FU-interval (in months)	/	107.89 (28.55)	/	104.09 (24.13)	/	126.50 (28.31)	0.163	-
**Clinical Characteristics**								
BDI-I	2.49 (2.29)	2.40 (2.59)	23.50 (11.67)	10.50 (8.86)	32.40 (5.68)	12.50 (8.36)	<0.001	HC < MDD HC < BD
HAM-D	/	1.71 (3.20)	/	6.34 (7.03)	/	6.00 (8.88)	0.004	HC < MDD HC < BD
YMRS	/	0.57 (1.04)	/	0.77 (0.92)	/	0 (0.00)	0.205	-
Number of hospitalizations	/	/	1.61 (1.46)	0.91 (1.51)	3.50 (1.98)	4.33 (6.95)	0.004	BD > MDD
Duration of hospitalization (in weeks)	/	/	4.65 (4.11)	2.18 (4.12)	15.08 (11.33)	11.00 (13.91)	0.001	BD > MDD
**Pearson’s Correlation with BDI-I**								
			*N* = 28	*N* = 28	*N* = 5	*N* = 6	-	-
BAG at Baseline (*z*-score)	-	-	−0.168 (*p* = 0.391)	−0.188 (p = 0.335)	−0.515 (*p* = 0.374)	0.281 (*p* = 0.588)	-	-
BAG at Follow-Up (*z*-score*)*	-	-	−0.130 (*p* = 0.510)	−0.214 (*p* = 0.274)	−0.031 (*p* = 0.960)	0.398 (*p* = 0.434)	-	-

Hospitalization data, data on patients with bipolar disorder and young mania rating scale are only available for the Münster site. Hamilton depression rating scale and young mania rating scale at baseline were unavailable for a majority of participants and are therefore not reported. *P*-values are reported for ANOVA with subsequent post hoc *t*-tests and between-subjects contrasts of a repeated measurements ANOVA if measurements at Baseline and Follow Up were available.

*HC* healthy controls, *MDD* major depressive disorder, *BD* bipolar disorder, *FU-interval* follow up interval, *BDI-I* beck depression inventory, *HAM-D* hamilton depression rating scale, *YMRS* young mania rating scale.

**Table 2:** Descriptive Statistics of the Hospitalization Sample.

	Healthy Controls N = 24		Hospitalized Patients N = 13		Not Hospitalized Patients N = 15	
	Baseline *M* (*SD*)	Follow Up *M* (*SD*)	Baseline *M* (*SD*)	Follow Up *M* (*SD*)	Baseline *M* (*SD*)	Follow Up *M* (*SD*)
BAG	2.51 (3.99)	−0.18 (4.03)	5.30 (5.86)	3.01 (5.61)	2.77 (5.32)	0.53 (5.44)
Age	29.8 (9.67)	45.83 (10.12)	37.62 (11.57)	47.31 (10.98)	32.40 (8.21)	42.47 (8.68)
Duration of FU (months)	-	125 (18.4)	-	117 (17.0)	-	121 (15.0)
BDI-I	2.72 (2.41)	3.43 (2.62)	29.00 (7.39)	15.60 (9.81)	16.38 (11.86)	4.92 (4.34)
HAMD	-	1.96 (3.66)	-	2.40 (3.29)	-	10.23 (10.32)
YMRS	-	-	-	0.73 (0.96)	-	0.46 (0.78)
Number of Hospitalizations	-	-	4.23 (1.64)	3.54 (4.63)	1.80 (1.61)	0 (0)

Some of the clinical values incorporate outliers and extreme values. Hamilton depression rating scale and young mania rating scale at baseline were unavailable for a majority of participants and are therefore not reported. BDI-I values at Baseline could not be derived for the entire sample.

*FU* follow-up interval, *BDI-I* beck depression inventory, *HAM-D* hamilton depression rating scale, *YMRS* young mania rating scale, *M* mean, *SD* standard deviation.

### MRI acquisition and preprocessing/VBM segmentation

Information on MRI acquisition, preprocessing and Voxel-Based Morphometry (VBM) segmentation can be found in Supplemental Material S1.

### Brain age gap

To calculate subject-specific brain age gaps, we used a previously developed brain age prediction model that was trained on a large representative sample of over 10,000 individuals of the GNC. The brain age model is based on a Monte-Carlo Dropout Composite-Quantile-Regression Neural Network (MCCQR-NN). For more details regarding the model architecture and training procedure, see [35]. In short, in contrast to existing brain age models, the MCCQR-NN model provides accurate estimations of predictive uncertainty in high-dimensional neuroimaging data while ensuring state-of-the-art model performance. It is therefore especially suited for the detection of subtle brain age changes, e.g., in clinical cohorts. All brain age predictions were made using the Python machine learning package PHOTONAI [36].

Brain age gaps are defined by the deviation between an individual’s chronological age and the age prediction provided by the brain age model. First, we fed unsmoothed gray matter voxel data to the model to retrieve brain age predictions. Second, to calculate brain age gaps, we subtracted the individual’s chronological age from the predicted age. Therefore, a positive BAG indicates accelerated while a negative BAG indicates decelerated brain aging.

As a single brain age prediction can vary in its predictive certainty, the MCCQR-NN additionally provides an estimation of the epistemic and aleatory uncertainty. This uncertainty estimation can be used to calculate uncertainty-corrected BAG z-scores common in normative modeling approaches [37]. Intuitively, correcting brain age predictions for their respective uncertainty pulls highly uncertain predictions towards the population norm, therefore reducing the risk of falsely classifying individuals as deviating from the normative distribution. For all analyses, we used uncertainty-corrected z-scores as primary measure. To make the interpretation easier, we used raw BAGs (measured in years) for all figures.

### Statistical analysis

We employed two multi-level linear-mixed effect models using the *nlme* function implemented in R [38] nested within the person controlling for age at baseline, site and length of the follow-up interval. In the first model, patients were categorized into a group according to their diagnosis (MDD or BD), further referred to as the *diagnosis model*. In the second model, limited to the Münster subsample, patients were categorized into a group according to their course of disease in the interval: patients with further hospitalizations between scans were categorized as “hospitalized patients”, while patients without further hospitalizations were categorized as “non-hospitalized patients”. We will further refer to this model as the *hospitalization model*. We then calculated between-subjects effects (main effect group and time) and a *group x time* interaction.

### Exploratory analysis

We conducted an exploratory analysis of variance (ANOVA) with the main effect of group (patients with hospitalizations until follow-up, patients without hospitalization until follow-up and HC) on BAG at baseline only, while controlling for age and length of follow-up interval. By excluding patients with bipolar disorder, controlling for medication intake and controlling for number of hospitalizations at baseline in a patient only analysis, we ensured the validity of our results in a secondary analysis (see “sensitivity analysis” and also Supplemental Material S3).

To follow up on our results, we explored predictive utility by conducting a logistic regression using the caret-package in R [39] predicting hospitalization during follow-up in the Münster sample using BAG (z-scores) at baseline while controlling for age at baseline, length of follow-up interval and diagnosis. We calculated predictive accuracy using 10-times repeated 10-fold cross-validation. This approach was chosen over leave-one-out cross-validation to reduce the variance of performance estimates, which is especially critical in small samples. Repeating the cross-validation ten times with different fold assignments further stabilized performance estimates and reduced sensitivity to specific data splits. Significance of the predictive model was assessed using a permutation test with 1000 permutations.

## Results

### Diagnosis effects

**Fig. 1:**
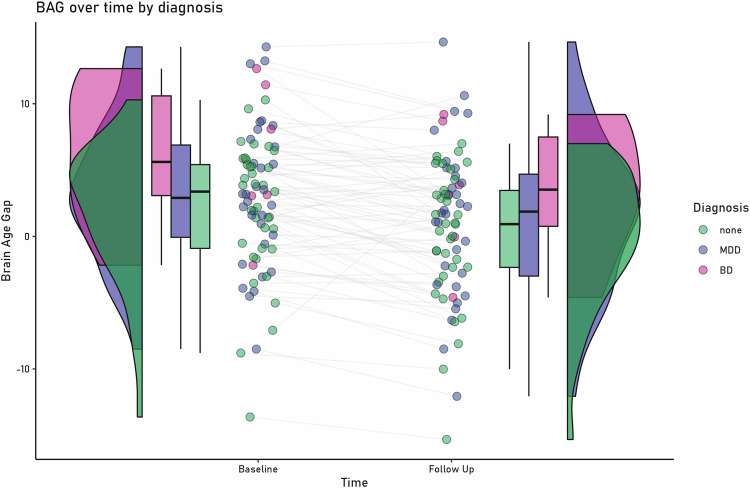
Change in brain age gap over time for the different diagnoses. Brain age gap is depicted in years, higher positive brain age gap indicates that the age was estimated higher than the actual age. MDD Major Depressive Disorder, BD Bipolar Disorder.

The mixed linear effects model yielded a significant main effect of diagnosis (χ^2^(10) = 7.527, p = 0.023): MDD patients differed from HC in their BAG (MDD > HC: t(75) = 2.202, p = 0.039; MDD: 2.27 + /- 5.68; HC: 1.00 + /- 5.12 years, d = −0.23), while BD showed a tendency to differ from HC (BD > HC t(69) = 1.868, p = 0.066; BD: 4.71 + /- 5.40 years); see also Fig. 1 and Table 1. BAG of BD and MDD patients did not differ (BD > MDD: t(69) = 9.693, p = 0.490), This effect was not modulated by time (χ^2^(12) = 0.448, p = 0.79). We found a main effect of time (χ^2^(8) = 32.082, p < 0.001) and age (χ^2^(5) = 29.934, p < 0.001) with BAG being lower for older participants (t(69) = −6.861, p < 0.001) and decreasing over time (t(72) = 3.611, p < 0.001). There was no correlation between BAG and acute illness severity measured with the BDI at baseline or follow up (all p’s > 0.27, see Table 1 for mean correlations).

### Effect of hospitalizations during the follow-up interval

**Fig. 2:**
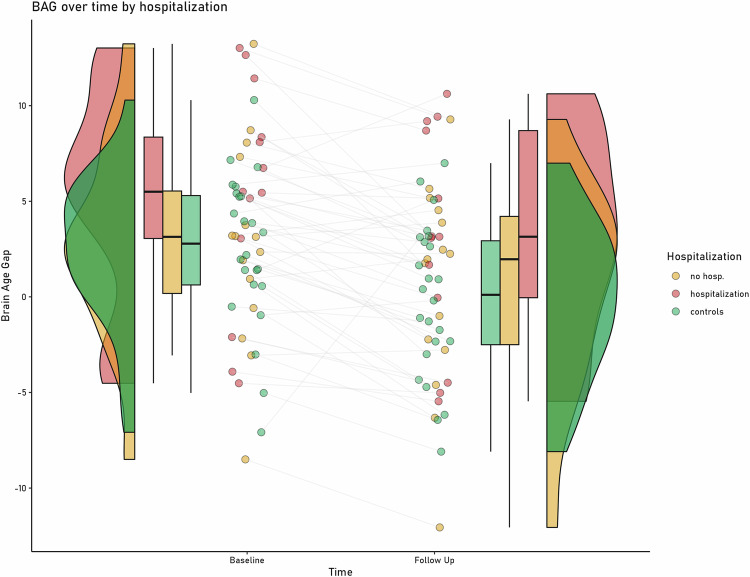
Change in brain age gap over time for different outcomes (hospitalization vs. no hospitalization in the 9-year interval). Brain age gap is depicted in years, higher positive brain age gap indicates that the age was estimated higher than the actual age.

The mixed-linear-effects model yielded a significant main effect of group (χ^2^(9) = 12.281, p = 0.002): While hospitalized patients showed a higher BAG than HC (t(47) = 3.403, p = 0.001; Hospitalized patients: 4.16 + /- 5.74; HC: 1.00 + /- 4.19 years, d = 0.62) and nonhospitalized patients (t(47) = −2.2827, p = 0.001; Nonhospitalized patients: 1.65 + /- 5.41 years, d = −0.45), nonhospitalized patients did not differ from HC (t(47) = 1.106, p = 0.27), see Fig. 2. This effect was not modulated by time (χ^2^(11) = 0.519, p = 0.77). Including either antidepressant intake at follow-up (yes/no) and excluding BD patients from the analysis led to the same pattern of results (see Supplement S3). Again, there was a significant effect of age at baseline (χ^2^(5) = 14.628, p < 0.001) and time (χ^2^(8) = 28.621, p <0.001). The direction of effect was comparable to the *diagnosis* model.

## Exploratory analysis

### Is BAG at baseline predictive of future hospitalizations during the follow-up interval?

The ANOVA model yielded a significant main effect of group on BAG at baseline (F(2,47) = 5.609, p = 0.001). BAG at baseline was significantly larger in hospitalized than in nonhospitalized patients and controls (t(47) = −3.242, p = 0.002), while nonhospitalized patients did not differ from controls in their BAG at baseline (t(47) = −0.667, p = 0.508).

The logistic regression revealed that BAG at baseline predicted hospitalizations during follow-up (z = 2.103, p = 0.035). However, the cross-validated mean test accuracy of 64.3% did not reach statistical significance (p = 0.071).

### Sensitivity analysis

BAG was not significantly different between men or women in our sample (Women: M = 2.03 years +/- 4.84 years; Men: M = 1.66 + /- 5.99 years; t(148) = 0.412, p = 0.681). A repetition of all analyses including sex as an additional covariate can be found in the Supplement S3a.

BAG was not significantly different between MDD and BD (t(68) = −0.806, p = 0.422). Hospitalized patients still differed from controls and nonhospitalized patients when excluding BD patients from the analysis (t(41) = −2.17, p = 0.035), while nonhospitalized patients did not differ from controls (t(41) = −1.00, p = 0.323). The difference between hospitalized and nonhospitalized patients also remained significant in a patient-only analysis (for details see Supplement S3b). BAG was not significantly different between medicated or unmedicated patients (t(23) = 1.060, p = 0.300). Our effects remained stable and statistically meaningful across all analysis when controlling for antidepressant medication intake (for details, see Supplement S3c).

BAG at baseline was also associated with the number of hospitalizations during follow-up while controlling for age and inter-scan interval (t(24) = 2.532, p = 0.018). The number of hospitalizations before baseline was also significantly predicting hospitalizations during follow-up (z = 2.294, p = 0.0218). Controlling for the number of hospitalization prior to our observation interval reduced the effect size of the prediction by BAG at baseline (Unadjusted Model: z = 2.103, adjusted model: z = 1.763). A partial Spearman correlation between BAG at baseline and the number of hospitalizations before baseline, controlling for age, revealed a significant positive association (ρ = 0.396, p = 0.040)

## Discussion

Using a state-of-the-art brain age prediction model trained on T1-weighted MRI images of 10,000 individuals, we calculated and compared brain age gaps in a longitudinal sample of 75 patients with affective disorders and healthy controls, measured at two time points with a mean follow-up length of nine years. BAG was higher in patients than in healthy participants. However, while it did not differ between diagnosis (BD and MDD), patients’ disease course was associated with BAG. Patients hospitalized within the follow-up interval showed a higher BAG than healthy controls, while patients without hospitalizations did not differ significantly from healthy participants.

In our study, we observe a correlation between age and BAG, with BAG estimates decreasing over time in all groups, indicated by a significant main effect of time. This can be attributed to the inherent correlation of BAG predictions with chronological age, a characteristic present in both the MCCQR-NN model used here and similar models reported in the literature [13, 35].

Although some studies show no difference in BAG between a healthy population and patients [23, 25, 40], our results are in line with a recent ENIGMA study showing differences between HC and MDD as well as a multi-modal brain age study showing differences between HC and BD [22, 41]. Thus, this provides further evidence for an association of psychopathology with the aging process of the brain.

Our observed BAG difference between MDD patients and healthy controls (approx. + 1.27 years, *d* = 0.23) falls within the lower range reported in large-scale studies (see, for example see [22] compared to [42]; for a review see Baecker et al., [34]).

There was no evidence of a group-by-time interaction, indicating that the BAG developed similarly within the groups over time, pointing toward a static effect of group (patient status, hospitalization status) at baseline and follow-up. Notably, hospitalized patients showed substantially higher BAG compared to both nonhospitalized patients and HC (Cohen’s *d* = 0.45 and d = 0.62), while nonhospitalized patients did not differ from controls. This suggests that elevated BAG is associated with recurrence in affective disorders, rather than being universally elevated across all patients with affective disorders.

In our exploratory post hoc analysis, BAG at baseline was also significantly associated with the number of prior hospitalizations. Adding this variable as a covariate to our logistic regression model predicting hospitalization during follow-up reduced the BAG effect size by 16% (from z = 2.103 to z = 1.763), indicating overlapping but non-redundant contributions. BAG did also not increase with subsequent hospitalizations, supporting its interpretation as a relatively stable marker, independent of increases in cumulative illness severity.

These findings suggest that BAG may partly reflect a trait-like vulnerability associated with recurrent illness courses. While this does not imply causality, it aligns with the view that BAG may capture latent risk for recurrence rather than acute disease state. This is further supported by the absence of correlations between BAG and symptom severity at baseline or follow-up (see also Table 1). Given the exploratory nature of these analyses and the limited sample size, findings should be interpreted with caution and warrant replication in larger samples.

If replicated, BAG could offer clinical utility by identifying individuals at elevated risk for illness recurrence, potentially informing more targeted preventive interventions. Similar clinical relevance has been demonstrated for BAG in early psychosis prediction, where a + 2.7 year BAG predicted future conversion to psychosis [43]. Our findings raise the possibility that BAG may likewise support early detection of recurrent trajectories in mood disorders (see [34] for further suggestion for the clinical use of BAG). Although our sample is relatively small, the effect sizes observed—particularly for hospitalized patients—suggest potential clinical utility. These applications will, however, require validation in larger and clinically diverse samples.

While in our exploratory logistic regression BAG at baseline significantly predicted hospitalization until follow-up, its cross-validation was not significant. One reason for this could be the limited sample size in this analysis (N = 28 patients were included). Larger studies should follow-up on our descriptive effects using predictive approaches.

In sum, the present study corroborates the idea of considering changes in gray matter in relation to the disease course of affective disorders. Both cross-sectional and longitudinal studies have already established a negative association between gray matter on the one hand and relapse, increased hospitalization, and episodes of illness on the other [27, 29, 44–50]. Our study supports and extends these findings: detrimental disease course manifests not only in local decreases in gray matter, e.g., hippocampus and insula [30, 51], but also in complex parameters based on gray matter measurements, such as BAG.

A higher brain age indicates that patients’ brains appear biologically older than their chronological age, with patients showing a recurrent disease course having a mean BAG at baseline of + 5.7 years. This accelerated brain aging [52, 53] may correspond to alterations of glucocorticoid release or changes in telomere length which have been associated with severity of depression [54, 55].

The early identification of patients with an increased risk for recurrent disease course is key to the development of preventive interventions, including the provision of social psychiatric support measures, the early use of more innovative psychopharmacological therapies, and the use of psychotherapy methods specifically for chronic patients, such as Cognitive Behavioral Analysis System of Psychotherapy [56–59]. However, despite an enormous amount of research, none of the previously identified putative biomarkers have found their way into clinical psychology or psychiatry [60]. Previous structural neuroimaging studies could not reveal that alterations of gray matter may predispose an individual to a recurrent disease course. To tackle this research gap, our longitudinal neuroimaging study, employing a machine learning approach, provides new insights into the relationship between the disease course of affective disorders and gray matter changes. A strength of this study lies in the combination of three innovative methodological aspects. First, it employs a multidimensional measure based on gray matter segments - the BAG. Second, this measure is based on a completely independent brain age model, trained on a substantial and representative German cohort exceeding 10,000 subjects. Third, the study’s longitudinal design explores patients with affective disorders over a large follow-up interval, allowing for a thorough characterization of the patient’s long-term disease course at baseline and during follow-up.

## Limitations

Due to the naturalistic nature of our longitudinal data, no causality can be inferred from our results. However, longitudinal neuroimaging studies are one important way to approach the hen-egg debate of gray matter alterations in affective disorders [45, 46]. Since our results are of correlative nature, we cannot rule out the possibility that not the disease course itself is causally related to the age-related gray matter alterations, but rather that other parameters predispose patients to this outcome, such as genetics, or a history of childhood maltreatment, bullying or increased self-blame [52, 53, 61, 62]. Our sample size was relatively small for our exploratory predictive analysis and the results should therefore be interpreted with caution. While our long follow-up interval offsets this limitation to some extent, replications of our findings are needed to increase generalizability of our findings.

In our analyses, we rigorously controlled for several potential confounders, including inter-scan interval, age, sex, medication, prior hospitalizations, and diagnostic group (BDD vs. MDD). However, the sample size was insufficient to model complex pharmacological regimens. We therefore controlled only for antidepressant medication intake, which limits the interpretability of potential effects related to other medication classes or polypharmacy.

While the cross-validated predictive accuracy of 64% significantly exceeds chance levels, its lack of statistical significance suggests a notable variability in the performance estimates across folds. To reliably assess the prognostic biomarker potential of BAG, a larger longitudinal sample will be necessary and should be investigated in future studies. In addition, while we focused on hospitalization as a clinically significant endpoint, longitudinal symptom trajectories may provide complementary insights into brain-aging processes and should be incorporated into future research.

## Conclusions

The present study revealed the brain age gap as a potential predictor for recurrence in affective disorders. In our analysis, a recurrent course of disease was associated with a larger BAG at baseline. This association was partly independent of the number of hospitalizations prior to our baseline measurements and medication intake at follow-up. Hence, a higher BAG may predispose patients to a poor outcome and hence could potentially be a relevant predictor of future disease course.

## Supplementary information

Supplemental material

## Data Availability

The data and code of this project are available on the open science framework (https://osf.io/qadxz/overview).

## Code availability

Data and reproducible scripts are available on OSF: https://osf.io/qadxz/.
