# Supplementary material for: MRI-derived estimation of biological aging in patients with affective disorders in a 9-year follow-up - a prospective marker of future recurrence
Source: Mol Psychiatry. 2025 Dec 14;31(5):2652–9. doi: 10.1038/s41380-025-03382-6 (PMC13099634; doi:10.1038/s41380-025-03382-6)
Supplement: Supplementary file 1 — Supplemental material [file 41380_2025_3382_MOESM1_ESM.docx]

**Supplement S1**

MRI preprocessing

Münster

T1-weighted high-resolution anatomical images of the head were acquired (Gyroscan Intera 3T, Philips Medical Systems, the Netherlands) at two time points (baseline and follow-up) using a three-dimensional fast gradient echo sequence (turbo field echo), repetition time = 7.4 ms, echo time = 3.4 ms, flip angle = 9°, two signal averages, inversion prepulse every 814.5 ms, acquired over a field of view of 256 mm (feet-head) x 204 mm (anterior-posterior) x 160 mm (right-left), frequency encoding in feet to head direction, phase encoding in anterior-posterior and right-left direction, reconstructed to voxels of 0.5 mm × 0.5 mm × 0.5 mm.

During preprocessing, 3 participants were excluded from further analysis due to movement artefacts at baseline or follow up leading to a final sample of N = 52 (BD = 6, MDD = 22, HC = 24) participants in Münster.

Dublin

During preprocessing, 3 participants were excluded from further analysis due to movement artefacts at baseline or follow up leading to a final sample of N = 23 participants (MDD = 10, HC = 13) in Dublin.

*VBM segmentation and data quality checks*

For VBM data, structural data was processed with the CAT12 toolbox (version 12.6 r1450, http://dbm.neuro.uni-jena.de/cat/) using SPM12 Matlab toolbox with default parameters. In brief, preprocessing steps included segmentation into grey matter, white matter, and cerebrospinal fluid and spatial normalization using the DARTEL algorithm [(Ashburner, 2007)](https://www.zotero.org/google-docs/?wfqZIL).

Data quality of VBM gray matter segments was verified by the “check homogeneity function” implemented in the CAT12 toolbox and outliers were visually inspected. In consequence, 6 subjects had to be removed from the sample retrospectively, 3 due to excessive head movement, 3 due to inadequate image quality resulting from other artifacts or strong noise.

Retest-reliability coefficients for each single subject were calculated by using the covariance structure of the gray matter segments of each individual baseline assessment with its corresponding follow-up image. This procedure was used for additional quality assurance in order to detect outliers as well as to confirm the correct labelling of pre-post image pairs.

### **Supplement S2. Sample characteristics for each site**

##### **Table 2. Sociodemographic, questionnaire and clinical data of study participants in Münster**

|  | **MDD (N=22)**  **M(SD)** | **BD (N=6)**  **M(SD)** | **HC (N=24)**  **M(SD)** | **P‐value according to χ 2‐tests or t‐tests between clinical groups** | **P‐value according to χ 2‐test or ANOVA between all groups** |
| --- | --- | --- | --- | --- | --- |
| Age at baseline | 35.1  (10.8) | 34  (7.5) | 29.8  (9.7) | .827 | .205 |
| Age at follow-up | 44.8  (10.7) | 44.3  (7.1) | 40.2  (10.5) | .918 | .297 |
| Interscan interval in months | 117.2  (14.8) | 126.5  (18.3) | 125.2  (18.4) | .207 | .233 |
| Sex (male/female) | 14/8 | 1/5 | 8/16 | .099 | .087 |
| Remitted at follow-up (yes/no) | 15/7 | 3/3 | - | .41 | - |
| hospitalized in interval (yes/no) | 9/13 | 4/2 | - | .262 | - |
| BDI at baseline | 18.9  (11.5) | 32.4  (5.7) | 2.7  (2.4) | .021^a^ | <.001^a^ |
| BDI at follow-up | 8.8  (9.3) | 12.5  (8.4) | 3.4  (2.6) | .396 | .005^a^ |
| HDRS at follow-up | 6.1  (8.3) | 6.0  (8.9) | 2.0  (3.7) | .991 | .107 |
| YMRS at follow-up | 0.8  (0.9) | 0.0  (0) | 0.6  (1) | .053 | .205 |
| Antidepressant medication at baseline  (yes/no) | 10/5 | 6/0 | - | .105 | - |
| Antidepressant medication at follow-up  (yes/no) | 8/14 | 4/2 | - | .184 | - |

MDD = patients with Major Depressive Disorder; BD= patients with Bipolar Disorder; HC = healthy controls; SD = standard deviation; BDI= Beck Depression Inventory; HDRS = hamilton depression rating scale; YMRS = young mania rating scale.

^a^Significant at statistical threshold P < 0.05.

##### **Table 3. Sociodemographic, questionnaire and clinical data of study participants in Dublin**

|  | **MDD (N=10)**  **M(SD)** | **HC (N=13)**  **M(SD)** | **P‐value according to χ 2‐tests or t‐tests between groups** |
| --- | --- | --- | --- |
| Age at baseline | 43.4  (7.9) | 40.9  (15.5) | .650 |
| Age at follow-up | 49.7  (8.3) | 47.2  (15.3) | .641 |
| Interscan interval in months | 75.2  (12) | 76  (9.9) | .862 |
| Sex (male/female) | 3/7 | 4/9 | .968 |
| Remitted at follow-up (yes/no) | 5/5 | - | - |
| BDI at baseline | 31.7  (6.6) | 2.1  (2.1) | <.001^a^ |
| BDI at follow-up | 13.3  (7.8) | 0.4  (0.7) | <.001^a^ |
| HDRS at follow-up | 7  (2.6) | 1.3  (2.1) | <.001^a^ |
| Antidepressant medication at baseline  (yes/no) | 9/1 | - | - |
| Antidepressant medication at follow-up  (yes/no) | 1/12 | - | - |

MDD = patients with Major Depressive Disorder; BD= patients with Bipolar Disorder; HC = healthy controls; SD = standard deviation; BDI= Beck Depression Inventory; HDRS = hamilton depression rating scale.

^a^significant at statistical threshold p < .05.

**Supplement S3. Sensitivity Analysis**

**S3a. Including Sex as an additional predictor**

1. *Diagnosis effects*

The mixed linear effects model still yielded a significant main effect of diagnosis (χ^2^(11) = 7.980, p = .023), while sex was not a significant predictor (χ^2^(6) = 0.085, p = .769): MDD patients differed from HC in their BAG (MDD > HC: t(68) = 2.140, p = .035) while BD showed a tendency to differ from HC (BD > HC t(68) = 1.868, p = .068). BAG of BD and MDD patients did not differ (BD > MDD: t(68)=-0.806, p =.423). This effect was not modulated by time (χ^2^(13) = 0.448, p = .79). We still found a main effect of time (χ^2^(9) = 32.082, p < .001) and age (χ^2^(5) = 29.934, p < .001, see results part of the manuscript for details).

1. *Effect of hospitalizations during the follow-up interval*

The mixed-linear-effects model yielded a significant main effect of group (χ^2^(10) = 13.432 , p = .001), while the predictor sex was not significant (χ^2^(6) = 0.361 , p = .547): While hospitalized patients showed a higher BAG than HC (t(46) = 3.563, p < .001) and nonhospitalized patients (t(46) = -2.301, p = .026), nonhospitalized patients did not differ from HC (t(46)= 1.282, p = .21). This effect was not modulated by time (χ^2^(12) = 0.519, p = .77). Again, there was a significant effect of age at baseline (χ^2^(5) = 14.628, p < .001) and time (χ^2^(8) = 28.621, p <.001). The direction of effect was comparable to the *diagnosis* model.

**Exploratory Analysis**

*Is BAG at baseline predictive of future hospitalizations during the follow-up interval?*

The ANOVA model yielded a significant main effect of group on BAG at baseline (F(2,46) = 6.288, p = .003), while sex was not a significant predictor (F(1, 46) = 2.073, p = .156). BAG at baseline was significantly larger in hospitalized than in nonhospitalized patients and controls (t(46) = -3.391, p = .001), while nonhospitalized patients did not differ from controls in their BAG at baseline (t(46) = 0.907, p = .520).

The logistic regression revealed that BAG at baseline predicted hospitalizations during follow-up (z = 2.100, p = .036), while sex was not a significant predictor (z = - 0.227, p = .821).

**S3b. Excluding bipolar patients from the analysis**

After excluding bipolar patients from the analysis, there was still a significant effect of hospitalization in our linear mixed model (χ²(9) = 7.632, p = .022). Hospitalized patients still differed from controls when excluding BD patients from the analysis (t(41) = , p = .035), while nonhospitalized patients did not differ from controls (t(41) = 1.223, p = .223). The difference between hospitalized and nonhospitalized patients also remained significant in a patient-only analysis (t(24) = 2.409, p = .024). The effect of hospitalization on BAG at baseline in the logistic regression also remained significant (z = 7.456, p = .024).

**S3c. Including Medication as an additional predictor**

To control for antidepressant medication intake, we conducted further analyses in the patient sample, also indicating our significant effect of hospitalization (χ²(9) = 7.632, p = .022), while the effect of medication was not significant (χ²(7) = 2.81, *p* = .093). The difference between hospitalized and not hospitalized patients in BAG while controlling for antidepressant medication intake was still marginally significant (*t*(23) = –2.05, *p* = .05). The inclusion of medication did not significantly improve model fit (χ²(7) = 2.81, *p* = .093), suggesting that medication exposure alone does not substantially explain variance in BAG. Importantly, there was still a significant main effect of hospitalization, even after including medication into the model (LRT: χ²(9) = 5.269, p = 0.021). In addition, BAG at Baseline was still associated with hospitalisation until follow-up (t(23) = -2.153 p = .042; z = 1.996, p = .046)), when controlling for antidepressant medication intake.
